# Integrative Analyses of Transcriptomics and Metabolomics in Immune Response of *Leguminivora glycinivorella* Mats to *Beauveria bassiana* Infection

**DOI:** 10.3390/insects15020126

**Published:** 2024-02-10

**Authors:** Hongqiang Fei, Juan Cui, Shiyu Zhu, Ye Xia, Yichang Xing, Yu Gao, Shusen Shi

**Affiliations:** 1 College of Plant Protection, Jilin Agricultural University, Changchun 130118, China; 20201556@mails.jlau.edu.cn (H.F.);; 2 Jilin City Academy of Agricultural Sciences, Jilin 132101, China; 3 Agriculture Science and Technology College, Jilin 132109, China

**Keywords:** *Beauveria bassiana*, *Leguminivora glycinivorella* Mats, RNA-Seq, metabolomics, immune response

## Abstract

**Simple Summary:**

*Leguminivora glycinivorella*, commonly known as the soybean borer, is one of the main pests in soybean production. *Beauveria bassiana* is an effective fungus for controlling the soybean borer. This study was based on soybean borer infected by *Beauveria bassiana*; combined transcriptome and metabolome analysis was performed. Three classes of antifungal differentially expressed genes were screened from the soybean borer, which were glutathione S-transferase (GSTs) genes, heat shock protein (HSP) genes, and cytochrome P450 (CYP450) genes. These three kinds of genes have an immune response in the pathway of glucose metabolism, lipid metabolism, and amino acid metabolism. These results provide a theoretical basis for improving the efficacy of *Beauveria bassiana* against soybean borer. According to the antifungal gene of soybean borer, we can develop a biological preparation to improve the control effect of *Beauveria bassiana*.

**Abstract:**

This study utilized *Beauveria bassiana* to infect *Leguminivora glycinivorella*, analyzed the effects on the transcriptome and metabolome, and further investigated the antibacterial function of *L. glycinivorella*. We performed transcriptome and metabolome sequencing on the *L. glycinivorella* infected with *B. bassiana* and its control groups, and performed a joint analysis of transcriptome and metabolome results. Upon screening, 4560 differentially expressed genes were obtained in the transcriptome and 71 differentially expressed metabolites were obtained in the metabolome. On this basis, further integration of the use of transcriptomics and metabonomics combined an analysis of common enrichments of pathways of which there were three. They were glutathione S-transferase (GSTs) genes, heat shock protein (HSP) genes, and cytochrome P450 (CYP450) genes. These three pathways regulate the transport proteins, such as ppars, and thus affect the digestion and absorption of sugars and fats, thus regulating the development of pests. The above conclusion indicates that *B. bassiana* can affect the sugar metabolism, lipid metabolism, and amino acid metabolism pathways of *L. glycinivorella*, and can consume the necessary energy, protein, and lipids of *L. glycinivorella*. The research on the immune response mechanism of pests against pathogens can provide an important scientific basis and target for the development of immunosuppressants. This study laid an information foundation for the application of entomogenous fungi to control soybean borer at the molecular level.

## 1. Introduction

*L. glycinivorella* Mats, commonly known as the soybean pod borer or small red worm, belongs to the insect class Lepidoptera, family Tortricidae, and genus Leguminivora [1]. From a global distribution perspective, soybean heartworm damage is primarily concentrated in East Asia including Russia, Japan, and North Korea [2,3,4]. Cultivated soybeans (*Glycine max*) are the primary hosts for *L. glycinivorella*. Additionally, *L. glycinivorella* are found in wild soybeans (*Glycine usurensis*) and *Sophora flavescens*. The host material used in this experiment was cultivated soybean provided by Jilin Agricultural University. *L. glycinivorella*, which are carnivorous insects, damage the growth of soybeans by burrowing their larvae into pods and feeding on seeds. These are a major global pest of soybean [5]. They are important pests in Northeast Asia and cause varying degrees of damage in different regions. The degree of damage varies depending on the year, with a general insect feeding rate of 10% to 15%. In more severe years, the insect feeding rate can reach 50% to 70%, resulting in a 20% to 40% reduction in soybean production [6,7,8].

Traditionally, the management of *L. glycinivorella* in agricultural production has depended on chemical control. However, chemical pesticides have negative effects on food safety, and their excessive application can harm the ecological environment. With the continuous deepening of research and changes in ideological concepts, the demand for environmentally friendly food and sustainable pest control is increasing. Biological control methods provide a solid foundation for future research [9,10,11,12,13]. This includes research on natural enemy insects, including predatory and parasitic enemies [14,15,16]. In addition to using the natural enemies of insects to control *L. glycinivorella*, the use of pathogenic microorganisms to control them is rapidly developing as an efficient and safe biological control method. *B. bassiana* is a widely used fungal insecticide, both domestically and internationally, and has been developed and utilized in several countries [17]. *B. bassiana* is commonly used to control *L. glycinivorella* during the period when larvae emerge from their pods and enter the soil. When the *L. glycinivorella* shed their pods, *B. bassiana* is mixed with the soil at a ratio of 1:25 and sprinkled onto the field. Studies have shown that reasonable use of *B. bassiana* can effectively prevent the occurrence of *L. glycinivorella* [18]. Therefore, it is crucial to elucidate the metabolic defense mechanisms of *L. glycinivorella* against *B. bassiana* infection.

Transcriptomics (RNA-Seq) has gradually emerged with the development of high-throughput sequencing technology, which is an important method for studying the function and structure of genes. Studying various genes in individuals, tissues, or cells under different conditions is an important aspect of genomics and includes a short sequencing time, high sequencing capacity, and low sequencing cost [19,20,21,22]. Metabolomics is an emerging technique in the field of omics that analyzes changes in metabolites produced by a cell or organism by identifying and quantifying them. This is an important component of system omics [23]. However, metabolomic technology still has significant differences in databases and cannot extract all metabolites using a single method. Compared to conducting a single metabolomics analysis, metabolomics is more frequently used in combination with various omics technologies, such as genomics and transcriptomics, to gain complementary advantages [24,25]. The screening and identification of genes involved in the immune response of insects to pathogenic fungi can facilitate the identification of different genes related to growth, development, immunity, pathogenicity, regulation, and metabolism, as well as the exploration of new molecular mechanisms. Pang et al. [26] identified a novel S-type arsenic strain symbiotic bacterium that can reduce the resistance of brown plant hoppers to imidacloprid. Through transcriptome and metabolome analyses, it was found that this strain infected brown plant hoppers, resulting in an increase in 19 metabolites and a decrease in 23 metabolites. The downregulation of biological metabolism may be the reason for the increased susceptibility to insecticides, which holds great potential for the development of agricultural pest control.

In order to explore the gene information related to immune response, such as pattern recognition, signal transduction, modulation, and defense response, during the infection of *B. bassiana* by soybean borer, this study conducted a combined analysis of transcriptomics and metabolomics to investigate the effects of *B. bassiana* on the gene expression and metabolic cycle of *L. glycinivorella*. Key differentially expressed genes involved in immune response in *L. glycinivorella* were screened, searching for the important target genes of soybean borer in response to *B. bassiana* infection. This provides a theoretical basis for the biological control of soybean borer. In addition, the quality and yield of soybean were improved from the aspect of pest control, removing the agricultural production of soybean borer control that mainly relies on chemical pesticides.

## 2. Materials and Methods

### 2.1. Test Strains and Insects

The *B. bassiana* strain used in this experiment was preserved and provided by the Key Laboratory of Soybean Disease and Pest Control at the Jilin Agricultural University. The tested *L. glycinivorella* were captured in the field at the soybean experimental base of Jilin Agricultural University.

### 2.2. Infection of L. glycinivorella with B. bassiana

Spore powder (1 g) was added to 1 L of sterile water containing 0.01% Tween-80, thoroughly shaken in a shaker, and a 1× spore suspension with a spore concentration of 108/mL was prepared. Mature *L. glycinivorella* were carefully placed into the prepared bacterial suspension using sterile tweezers and allowed to remain for 3 s to ensure full contact with the suspension. Mature larvae were placed on filter paper to absorb any excess bacterial suspension from the surface of their bodies. Mature larvae were immersed in sterile distilled water containing Tween-80 for 3 s as the control group (CK). The bodies of infected insects from each treatment group were placed in a 50 mL centrifuge tube filled with sterile soil, with 50 heads per treatment group and three replicates. The treatment groups were set up as follows: Groups A, B, and C. In Group A, the parasite was collected 32 h after infection; in Group B, the parasite was collected 64 h after infection; and in Group C, the parasite was collected 96 h after infection. The samples were stored at −80 °C for subsequent transcriptomic and metabolomic analyses.

### 2.3. Extraction and Transcriptome Sequencing of Total RNA from Soybean Heartworm

Total RNA was isolated from the soybean heartworm samples using a total RNA isolation kit. For the cDNA library on the Illumina sequencing platform (HiSeq)™, sequencing was performed using 2500 and HiSeq X Ten. The quality of the raw sequencing data was evaluated using FastQC, and Trinity was used to mix and concatenate the valid read data of the samples to obtain information on the unique sequence [27,28]. HISAT2 (2.1.0) software was used to calculate the mapping fragment per thousand bases per million (FPKM) values of exons and evaluate gene expression. For samples with biological replicates, DESeq was used to identify differentially expressed genes (DEGs). The screening criteria for significantly different genes were set as q Value ≤ 0.05 and|log2FoldChange| ≥ 1 [29,30,31]. We conducted Gene Ontology (GO) classification and a Kyoto Encyclopedia of Genes and Genomes (KEGG) metabolic pathway enrichment analysis of DEGs to study their distribution in the annotation function and to elucidate the differences in gene function between samples.

### 2.4. Widely Targeted Metabolomic Analysis of L. glycinivorella

We conducted an extensive targeted metabolomics analysis to investigate the changes in the accumulation of related immune metabolites in *L. glycinivorella* under different infection times of *B. bassiana*. Metabolite analysis of soybean heartworm samples was performed by Shanghai Shenggong Biotechnology Co., Ltd. (Shanghai, China). Principal component analysis (PCA) and orthogonal projection latent structure discriminant analysis (OPLS-DA) were used to analyze differences in metabolites between samples [32]. Variable importance projection (VIP, version 1.6.2) of the OPLS-DA model was used to screen for differential metabolites. Metabolites with a Fold Change ≥ 2 or ≤0.5 and VIP ≥ 1 were considered differentially accumulated metabolites (DAMs). The accumulation of metabolites in *L. glycinivorella* at various infection times was analyzed and compared using the ropls R software package (version 1.6.2) and PCA [33,34,35]. The data were normalized, and heatmaps were created to cluster all samples for better visualization.

### 2.5. Combined Analysis of the Transcriptome and Metabolome of Soybean Heartworm

The DEGs and DAMs in pathways formed by various control combinations were analyzed based on metabolite content and gene expression values in *L. glycinivorella* after different infection times. First, we analyzed the DEGs and DAMs related to synthesis using pathway analysis. To investigate the relationship between the transcriptome and metabolome, we mapped DEGs and DAMs to the KEGG pathway database to obtain common pathway information for both datasets [36,37].

### 2.6. Real-Time Fluorescence Quantitative PCR Detection

Fourteen DEGs for real-time fluorescence quantitative PCR (qRT-PCR) analysis and validation were randomly selected: DN49208_ C1_ G5, DN56089_ C1_ G3, DN58084_ C0_ G3, DN57940_ C2_ G4, DN57940_ C2_ G1, DN60066_ C0_ G1, DN55598_ C1_ G1, DN58307_ C0_ G1, DN58307_ C0_ G3, DN58574_ C2_ G1, DN58574_ C2_ G6, DN60415_ C1_ G1, DN62201_ C1_ G2, and DN62201_ C1_ G1. cDNA was extracted from the *L. glycinivorella* at each stage of infection. Agilent Technologies Strategy M × 3000P and SYBR pre-mixed Ex Taq were used according to the manufacturer’s instructions. For the internal reference gene, 18 s was used. Relative expression levels were calculated using the 2^−∆∆Ct^ method. The primers used in this study are listed in Table S1 (attachment).

## 3. Results

### 3.1. Transcriptomic Analysis of the Immune Response of Soybean Heartworm Infected with B. bassiana

#### 3.1.1. Quality Control of Samples and Analysis of Differences in Gene Expression

Sequencing libraries were constructed under processing and control conditions for the 12 soybean heartworm samples, resulting in 67.6 Gb of clean data. The Q30 base percentage of all samples was ≥93.54%, indicating the reliability of the data. The GC content of all samples was approximately 49.49%, indicating a high sequencing accuracy. More than 90% of the reads were specifically aligned to the reference genomes in transcriptome assay samples. The high specific alignment rate indicated that the next step in transcriptome data analysis could be performed, confirming the accuracy of the transcriptome data and enabling further analysis.

**Figure 1:**
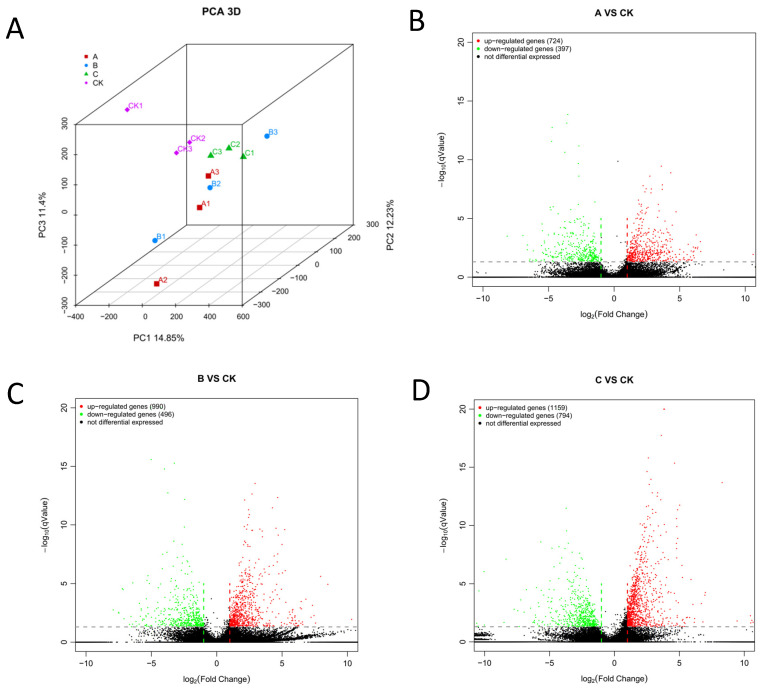
PCA analysis of samples and volcano plot of differentially expressed genes. (**A**) PCA plots between samples of A, B, and C treatment groups and CK control group. A treatment was *B. bassiana* infection for 32 h, B treatment was *B. bassiana* infection for 64 h, C treatment was *B. bassiana* infection for 96 h; set 3 replicates per process. (**B**) The gene expression level of the larvae at 32 h after infection (A vs. CK). (**C**) The gene expression level of the larvae at 64 h after infection (B vs. CK). (**D**) The gene expression level of the larvae at 96 h after infection (C vs. CK). Green points: downregulated genes; red points: upregulated genes; black points: genes with insignificant changes in expression. Dot line: threshold to decide whether the gene’s expression level changes observably.

To evaluate the reproducibility of the transcriptome data from the *L. glycinivorella*, we conducted PCA on 12 samples, as shown in Figure 1A. The first, second, and third axes explained 14.85%, 12.23%, and 11.4% of the total variation, respectively, whereas they collectively explained 38.48% of the total variation. Among them, A1, A2, and A3 were grouped together; B1, B2, and B3 were grouped together; and C1, C2, and C3 were grouped together. A1, A2, and A3 were three replications of *B. bassiana* infection for 32 h. B1, B2, and B3 were three replications of *B. bassiana* infection for 64 h. C1, C2, and C3 were three replications of *B. bassiana* infection for 96 h. The samples from the same group had a relatively concentrated spatial distribution. By screening the conditions q < 0.05 and |log2Fold Change| > 1, 4560, DEGs were identified between the soybean heartworm treatment and control groups, of which 2873 were upregulated and 1687 were downregulated, as shown in Figure 1B–D.

#### 3.1.2. GO Enrichment Analysis of Differentially Expressed Genes

**Figure 2:**
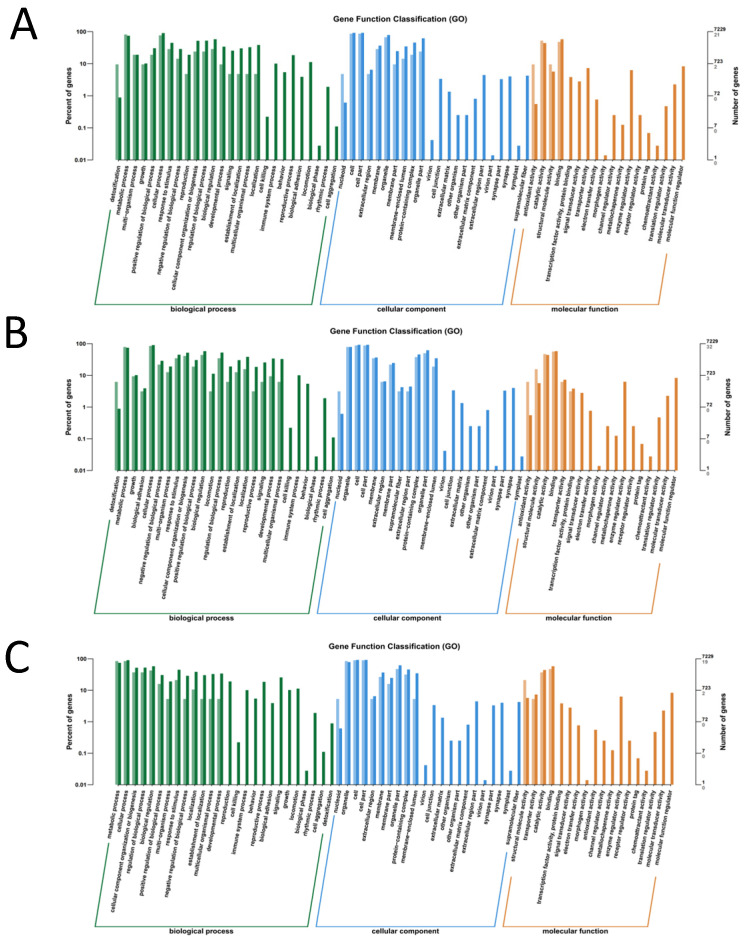
GO enrichment analysis of differentially expressed genes. (**A**) The larvae were infected by *B. bassiana* for 32 h (A vs. CK). (**B**) The larvae were infected by *B. bassiana* for 64 h (B vs. CK). (**C**) The larvae were infected by *B. bassiana* for 96 h (C vs. CK).

GO functional annotation of the genes included biological processes (BP), cellular components (CC), and molecular functions (MF). The DEGs in the A vs. CK group were categorized into 66 GO terms (Figure 2A). BP categories were mainly distributed in metabolic processes, cellular processes, biological regulation, cellular tissue components or biogenesis, and responses to stimuli. The MF category was mainly distributed in terms of binding activity, molecular structural activity, etc. The CC category was mainly distributed in cells and cellular components, organelle components, and protein complexes. The DEGs in the B vs. CK group were divided into 66 GO terms (Figure 2B). The BP category was mainly distributed in cellular processes, metabolic processes, cellular component organization or biogenesis, and biological regulation. The MF category was mainly distributed in binding activity, catalytic activity, etc. The CC category was mainly distributed in cells, cellular components, organelle components, etc. The DEGs in the C vs. CK group were divided into 65 GO terms (Figure 2C). The BP category was mainly distributed in metabolic processes, cellular processes, and biological regulation. The MF category was mainly distributed in catalytic activity, binding activity, and molecular structural activity, whereas the CC category was mainly distributed in cellular and organelle components. According to the GO classification, we found that the expression of differentially expressed genes was concentrated in the cellular and organelle components after the infection, and it also activated a series of self-regulation and metabolism to resist the invasion of bacteria.

#### 3.1.3. KEGG Pathway Analysis of Differentially Expressed Genes

**Figure 3:**
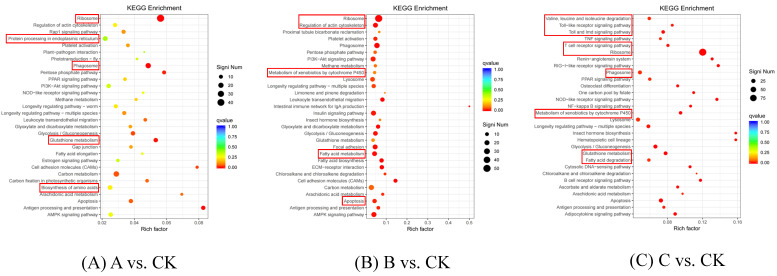
Bubble diagram of differentially expressed gene KEGG pathway. (**A**) The larvae were infected by *B. bassiana* for 32 h (A vs. CK). (**B**) The larvae were infected by *B. bassiana* for 64 h (B vs. CK). (**C**) The larvae were infected by *B. bassiana* for 96 h (C vs. CK). The size of Qvalue is indicated by the color of the dot, and the smaller the Qvalue, the closer the color is to red; the number of distinct genes contained in each function is represented by the size of the dots. The main enrichment pathway is in the red box.

Gene expressions often function together to regulate specific functions in the plant body. To identify the pathways significantly regulated during fungal invasion, we used *L. glycinivorella* infected with *B. bassiana* as a research model. We compared the significant DEGs in *L. glycinivorella* at different infection time points using the KEGG pathway and further analyzed the functions of these significant DEGs in the relevant pathways. DEGs in the three comparative groups (A vs. CK, B vs. CK, and C vs. CK) were enriched in 188, 203, and 192 metabolic pathways, respectively. We considered a significance level of *p* < 0.05 as the significance criterion for enrichment analysis and presented the top 20 pathways with their significance rankings in a bubble scatter plot, as depicted in Figure 3. These pathways were significantly regulated by the induction of fungal infection.

Further analysis showed that most of the differential genes were enriched in the pathways related to insect self-growth and resistance, and that among the 188 A vs. CK pathways, the pathways with the highest number of DEGs were glutathione metabolism, ribosome pathway, phagosome pathway, protein processing in the endoplasmic reticulum, and amino acid biosynthesis. We identified three types of upregulated DEGs related to insect immunity: glutathione S-transferase (GST) genes, heat shock protein genes, and cytochrome P450 class motifs. These results indicated that infection with *B. bassiana* successfully induced an immune defense response in *L. glycinivorella*. Among the 203 pathways of B vs. CK, the main enriched pathways of DEGs were ribosomes, cytochrome P450 metabolism of exogenous substances, regulation of actin cytoskeleton, fatty acid metabolism, and cell apoptosis. We found that the three types of immune-related genes upregulated in the A vs. CK group also showed the same upregulation trend in B vs. CK, indicating that after 64 h of infection, *L. glycinivorella* still produced immune-related proteins and regulated the apoptosis of damaged cells. Among the 192 pathways of C vs. CK, the main enriched pathways of DEGs were ribosomes, P450 cytochrome P450 metabolism of exogenous substances, glutathione metabolism, T cell receptor signaling pathway, phagosomes, amino acid biosynthesis, fatty acid metabolism, protein processing in the endoplasmic reticulum, regulation of actin cytoskeleton, peroxidase metabolism, and purine metabolism. The DEGs that were upregulated during 32 h and 64 h of infection underwent expression changes after 96 h of infection, and GST-like genes changed from upregulated to downregulated. This indicates that after 96 h of infection, the immune response of *L. glycinivorella* to *B. bassiana* still existed, and because of the longer infection time, the damage to insect body functions was aggravated and some genes that were originally upregulated and involved in immunity were weakened. T cells play a role in combating antigens that enter the insect body and enhance the immune function. In addition, insect cells remove toxic substances produced in the body and reduce the production of peroxides.

### 3.2. Metabolomic Analysis of the Immune Response of L. glycinivorella after Infection with B. bassiana

#### 3.2.1. Quality Control of Metabolome Samples

**Figure 4:**
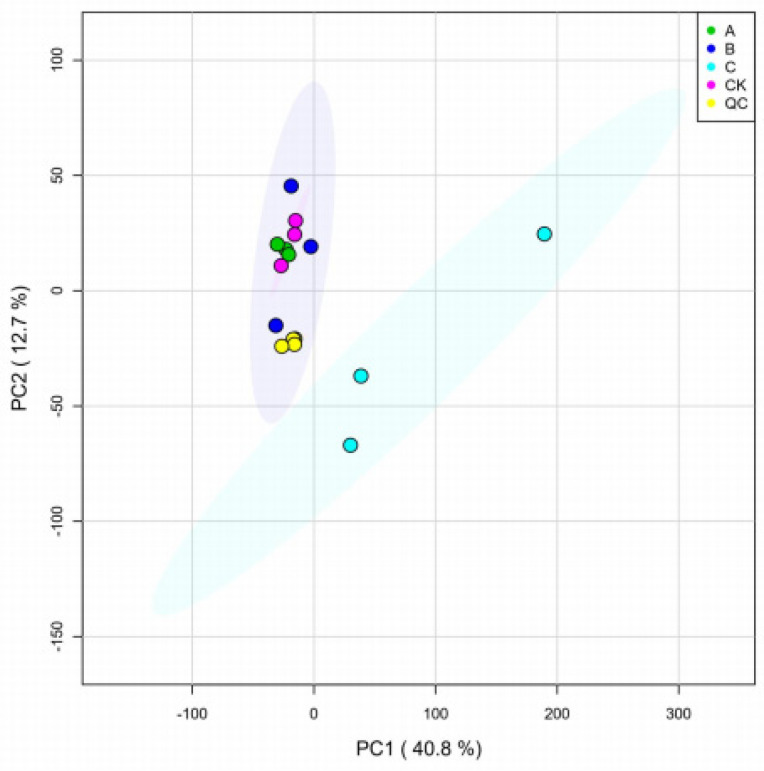
Differential metabolite PCA analysis. PCA plots between samples of A, B, and C treatment groups and CK control group. The A treatment was *B. bassiana* infection for 32 h, B treatment was *B. bassiana* infection for 64 h, and C treatment was *B. bassiana* infection for 96 h; set 3 replicates per process.

Quality control PCA of metabolome samples is an unsupervised method for pattern recognition and multidimensional statistical data analysis. The analysis results showed a trend of metabolome separation between the groups, indicating whether there were differences in the metabolome between sample groups. OPLS−DA is a multivariate statistical analysis method that uses a supervised pattern recognition function. Compared to PCA, OPLS−DA maximizes inter-group differentiation and facilitates the search for differential metabolites. PCA and OPLS−DA analyses were performed on the two samples and it was found that there was a clear distinction between the groups for each sample, indicating the presence of differential metabolites between the samples, as shown in Figure 4.

#### 3.2.2. Screening of Differentially Expressed Metabolites Related to Antibacterial Activity

Based on the OPLS−DA results, differential metabolites between the two samples were preliminarily screened using the VIP values. Further screening of differential metabolites based on p-values and fold changes resulted in the identification of 71 differential metabolites, including 44 upregulated and 27 downregulated ones. These metabolites included organic acids, amino acids, alkaloids, flavonoids, phenolic acids, lipids and their derivatives, nucleotides and their derivatives, and terpenes. Among these, organic acid metabolites were the most abundant, whereas terpenoid metabolites were the least abundant.

#### 3.2.3. KEGG Enrichment of the Differential Metabolites

**Figure 5:**
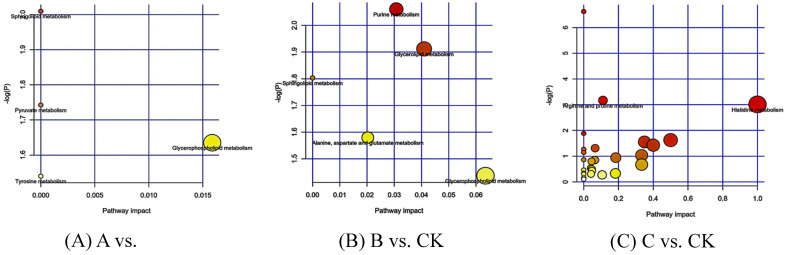
KEGG enrichment analysis of differential metabolites. (**A**) The larvae were infected by *B. bassiana* for 32 h (A vs. CK). (**B**) The larvae were infected by *B. bassiana* for 64 h (B vs. CK). (**C**) The larvae were infected by *B. bassiana* for 96 h (C vs. CK). Each bubble represents a metabolic pathway, and the horizontal coordinates of the bubble and the bubble size indicate the influence factor size of the pathway in the topology analysis The vertical coordinates of the bubble and the color of the bubble represent the P value of the enrichment analysis (take negative natural logarithm, that is, -ln (p)). The darker the color, the smaller the P value and the more significant the enrichment degree.

KEGG pathway enrichment analysis was conducted on 71 differential metabolites, revealing that they were most enriched in various metabolite types, including glycerophospholipid, sphingosine, pyruvate, tyrosine, skimming, glyceride, lipoprotein, and amino acid metabolism alanine, arginine, proline, histidine, aspartic acid, and glutamic acid (Figure 5).

### 3.3. Transcriptome and Metabolome Analysis of Immune Response in L. glycinivorella Infected with B. bassiana

#### 3.3.1. Cluster Analysis of Differentially Expressed Genes and Metabolites

**Figure 6:**
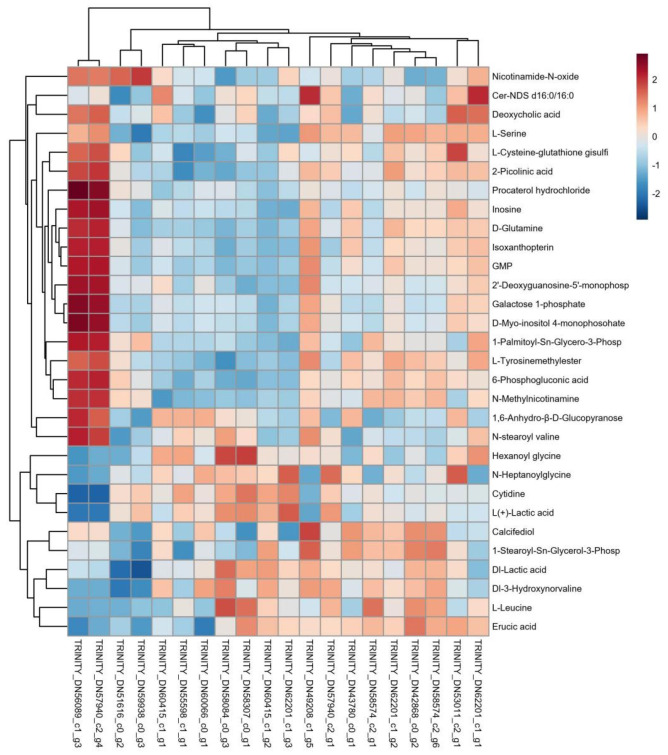
Cluster heatmap of differentially expressed genes and metabolite correlation. The correlation clustering heatmap results indicate that the red part represents a positive correlation between differentially expressed genes and metabolites, while the green part represents a negative correlation between differentially expressed genes and metabolites.

A further correlation analysis was conducted on the DEGs and metabolites, revealing associations between the 20 DEGs and 30 differentially expressed metabolites. Pearson correlation coefficient calculations were performed on these DEGs and metabolites, and a correlation clustering heatmap was constructed (Figure 6).

#### 3.3.2. Analysis of Antibacterial-Related Differential Genes and Metabolite Regulation Mechanisms

**Figure 7:**
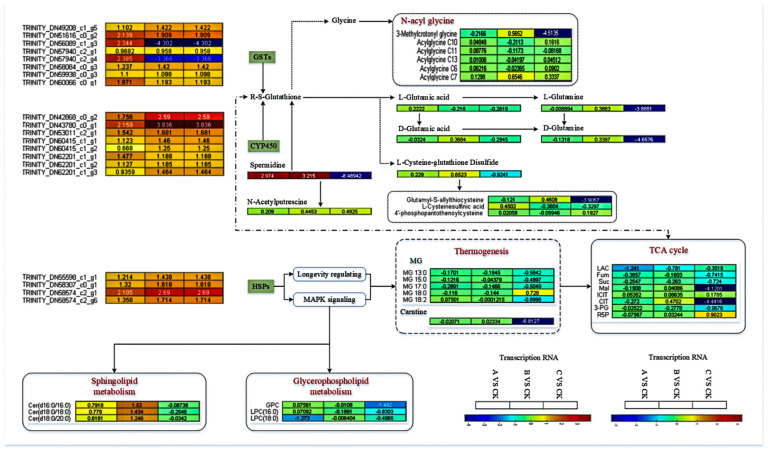
The regulatory mechanism of antimicrobial differentially expressed genes and metabolites. The relationships between GSTs, CYP450, HSPs, and downstream metabolites were demonstrated. Red represents a positive correlation and blue represents a negative correlation; the stronger the correlation, the darker the color.

Differential genes and metabolites were analyzed, and a metabolic pathway diagram was drawn by integrating the changes in soybean heartworm infection with *B. bassiana*. Glutathione S−transferase, heat shock protein, and cytochrome P450 were the critical pathways in the animal response to stress. Figure 7 shows the changes in the DEGs and metabolites within the three pathways. Glutathione S−transferase class genes were mainly involved in eight genes. The heat shock protein class genes were mainly involved in four genes. Cytochrome P450 class genes were mainly involved in eight genes. These results indicate that the *B. bassiana* infection successfully induced the immune defense response in *L. glycinivorella*.

### 3.4. qRT-PCR Validation of Transcriptome Sequencing Data

**Figure 8:**
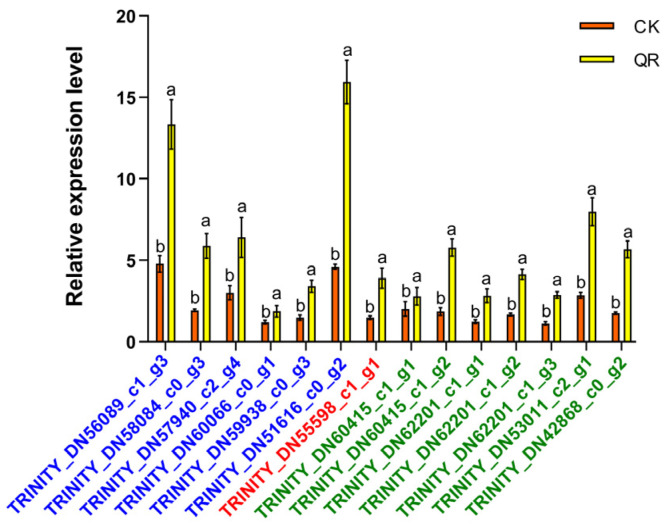
Comparison of differentially expressed gene transcriptome and qRT-PCR results. QR is the larva infected by *B. bassiana*, CK is the larva not infected by *B. bassiana*. The 14 genes included GSTs (the gene number is blue), CYP450 (the gene number is red), and HSPs (the gene number is green). The 18s gene was used as an internal reference gene. The data are represented as the mean ± S.D. (n = 3); different lowercase letters indicate significant difference at 0.05.

To validate the authenticity of the transcriptome sequencing results, we selected 14 genes with differential expressions. These included six GST, one HSP, and seven CYP450 genes. The selected genes that were verified exhibited significantly upregulated expression. qRT-PCR was used to confirm the transcription levels in the experimental tissues. The results showed that the larvae infected with *B. bassiana* in the treatment group (QR) exhibited significant differences from those in the control group (CK). Additionally, the relative expression levels of the six GST genes increased by 175%, 205%, 113%, 54%, 129%, and 246%, respectively. The relative expression of class 1 HSP genes increased by 163%. The relative expression levels of the seven CYP450 genes increased by 38%, 212%, 128%, 147%, 154%, 178%, and 224%, respectively. The trend observed in the transcriptome sequencing data was consistent with the trend in DEGs validated by qRT-PCR (Figure 8), suggesting that transcriptome sequencing data are more reliable.

## 4. Discussion

This study investigated the influence of *B. bassiana* on gene expression and metabolic differences resulting from soybean heartworm infection, using a combined analysis of the transcriptome and metabolome. The advantage of biological control is that it is non-toxic, non-polluting, and environmentally friendly, aligning with the concept of green production. Biological control of agricultural pests lasts longer than chemical control and has a minimal impact on agro-ecology. A total of 2873 upregulated and 1687 downregulated genes were identified, along with 44 upregulated and 27 downregulated differential metabolites. This indicates that *B. bassiana* infection causes significant changes in gene expression and metabolite profiles in soybean heartworms.

GO functional analysis of DEGs revealed that they were mainly enriched in cellular processes, metabolic processes, binding activity, catalytic activity, cellular and cellular components, and cellular and organelle components. KEGG enrichment analysis of DEGs showed that they were mainly enriched in arginine and glycine, glutamine, and sphingolipid metabolism pathways. Among them, the glutathione S-transferase (GST) genes, heat shock proteins (HSPs), and cytochrome P450 motifs were the most significant, and these genes were involved in most insect immune regulatory responses. By relying on body fluids and cells to recognize receptors and activate immune pathways, fat bodies and blood cells produce and secrete antibacterial factors [38,39,40], which exert immune effects. GST plays a crucial role in the defense systems of organisms, often exerting antioxidant and detoxification functions to protect organisms under stress [41,42,43,44,45]. HSPs are a class of stress proteins. HSPs can be induced by various stressors, helping to alleviate the damage caused by stress and facilitating repair [46,47,48,49]. Cytochrome P450 is a metabolic enzyme widely present in animals and plants and plays an important role in the growth, development, and drug resistance of insects [50,51,52,53]. The remaining differentially expressed genes were mainly enriched in the ribosome, phagosome, amino acid biosynthesis, fatty acid metabolism, and protein processing pathways in the endoplasmic reticulum, indicating that infection of *L. glycinivorella* with *B. bassiana* may consume their energy metabolism and compete with their development. The pathways enriched by differential metabolite KEGG functions were involved in the metabolism of alanine, arginine, proline, histidine, aspartic acid, and glutamic acid. These pathways were related to amino acid metabolism, which is consistent with the KEGG enrichment results of the transcriptome.

Transcriptome metabolomic analysis revealed enrichment in three pathways, among which the P450 pathway and glutathione transferase pathway were upstream pathways that affected glutathione content in insect serum, consequently affecting the metabolism of downstream arginine and glycine. Glycine metabolism directly affects changes in the lipid compound N-acetyl-glycine, whereas changes in arginine cause changes in its metabolites. In addition, the transformation of glutathione causes changes in the levels of glutamic acid, cysteine, and glutamine, consequently affecting the levels of certain amino acid metabolites. The heat shock protein gene is located upstream of the MAPK and lifespan regulation pathways and can regulate the metabolism of multiple lipid pathways, including glycerophospholipid metabolism, sphingolipid metabolism, lipid absorption and synthesis, and thermogenesis. The thermogenic effect is the pathway that has the most significant impact on HSPs. The thermogenic effect and action of p450 can significantly impact the TCA cycle pathway, thereby affecting the entire respiratory process and contributing to the development of pests. In addition, the aforementioned three types of pathways regulate transport proteins, such as PPARs, thereby affecting the digestion and absorption of sugars and fats and exerting regulatory mechanisms on pest development. These results indicate that *B. bassiana* infection can disrupt sugar metabolism, amino acid metabolism, and lipid metabolism pathways of *L. glycinivorella*, depleting energy, lipids, and proteins in their bodies. The main challenge of this experiment was to acquire soybean borer and deal with unpredictable natural environments. These factors may limit the duration of the pilot study and make it more challenging. Furthermore, in future use, the infection effect of *B. bassiana* that is directly related to the outdoor and moderate temperature should be studied, as the weather is also an important factor.

## 5. Conclusions

In this study, we investigated the effects of *B. bassiana* infection on gene expression and metabolism of *L. glycinivorella* through a combination of transcriptome and metabolome analyses. Three candidate genes, glutathione S-transferase, heat shock protein, and cytochrome P450, were found to be highly expressed during the immune response. The immune response pathways of the three candidate genes included arginine and glycine, L-glutamine, sphingolipid, glucose, lipid, and amino acid metabolic pathways. The results indicated that the gene expression and metabolites of *B. bassiana* exhibited significant changes after infection with *L. glycinivorella*. Fourteen candidate genes were screened, and all showed upregulation and significant expression in the three infection comparison groups. To clarify the immune response mechanism of *L. glycinivorella* after *B. bassiana* infection, this study offers valuable insights into the molecular mechanism of the immune response in *L. glycinivorella*, providing a theoretical foundation and new idea for biological control.

## Data Availability

The transcriptomic and metabolomic raw data related to this thesis have been uploaded to NCBI (https://dataview.ncbi.nlm.nih.gov/object/PRJNA908298) under the accession number SUB12334170. Accessed on 23 January 2023.

## Supplementary Materials

The following supporting information can be downloaded at: https://www.mdpi.com/article/10.3390/insects15020126/s1, Table S1: Primers for qRT-PCR.
